# Artesunate enhances the therapeutic response of glioma cells to temozolomide by inhibition of homologous recombination and senescence

**DOI:** 10.18632/oncotarget.11972

**Published:** 2016-09-12

**Authors:** Nancy Berte, Stefanie Lokan, Marcus Eich, Ella Kim, Bernd Kaina

**Affiliations:** ^1^ Department of Toxicology, Medical Center of University of Mainz, D-55131 Mainz, Germany; ^2^ Department of Neurosurgery, Medical Center of University of Mainz, D-55131 Mainz, Germany

**Keywords:** glioblastoma, temozolomide, artesunate, DNA repair, senescence

## Abstract

Glioblastoma multiforme (GBM), a malignant brain tumor with a dismal prognosis, shows a high level of chemo- and radioresistance and, therefore, attempts to sensitize glioma cells are highly desired. Here, we addressed the question of whether artesunate (ART), a drug currently used in the treatment of malaria, enhances the killing response of glioblastoma cells to temozolomide (TMZ), which is the first-line therapeutic for GBM. We measured apoptosis, necrosis, autophagy and senescence, and the extent of DNA damage in glioblastoma cells. Further, we determined the tumor growth in nude mice. We show that ART enhances the killing effect of TMZ in glioblastoma cell lines and in glioblastoma stem-like cells. The DNA double-strand break level induced by TMZ was not clearly enhanced in the combined treatment regime. Also, we did not observe an attenuation of TMZ-induced autophagy, which is considered a survival mechanism. However, we observed a significant effect of ART on homologous recombination (HR) with downregulation of RAD51 protein expression and HR activity. Further, we found that ART is able to inhibit senescence induced by TMZ. Since HR and senescence are pro-survival mechanisms, its inhibition by ART appears to be a key node in enhancing the TMZ-induced killing response. Enhancement of the antitumor effect of TMZ by co-administration of ART was also observed in a mouse tumor model. In conclusion, the amelioration of TMZ-induced cell death upon ART co-treatment provides a rational basis for a combination regime of TMZ and ART in glioblastoma therapy.

## INTRODUCTION

Treatment of the highly aggressively growing brain tumor glioblastoma multiforme (glioma WHO grade IV; GBM) is usually not effective, and patients have a dismal prognosis with a median survival of 14.6 months [1]. Treatment consists of resection of the tumor followed by radio- and chemotherapy [1]. First line drug in chemotherapy is the methylating agent temozolomide (TMZ) [2], which induces a dozen DNA adducts [3]. Among these, the minor adduct *O^6^*-methylguanine (*O^6^*-MeG) represents the most severe killing lesion, provided it is not repaired by *O^6^*-methylguanine-DNA methyltransferase (MGMT) [4]. Mispairing of *O^6^*-MeG with thymine during DNA replication leads to *O^6^*-MeG/T mismatches that are recognized by the mismatch repair system (MMR), which then performs erroneous repair cycles [5, 6]. Secondary lesions, most probably extended gaps, are created during this faulty repair process, leading to blockage of DNA replication in the next replication cycle, which in turn gives rise to DNA double-strand breaks (DSB) [7, 8]. These DSB trigger cell death by apoptosis, which we have shown is effectively induced in glioma cells as a late response following TMZ treatment [9]. In addition to apoptosis, it was shown that TMZ also induces autophagy and senescence in glioma cells [10–12]. Thus, identifying agent(s) that inhibit pro-survival pathways such as autophagy and replicative senescence and foster cell death pathways is highly desired and anticipated to support brain tumor therapy.

Artesunate (ART) is a semi-synthetic derivative of the herbal *Artemisia annua* ingredient artemisinin, which was extensively used for centuries in traditional Chinese medicine (TCM) and is currently being used as antimalarial drug because of its potent activity against the chloroquine resistant pathogen *Plasmodium falciparum* [13]. It is a natural endoperoxide that forms intracellular reactive oxygen species (ROS) [14]. ART was shown to exert cytotoxic activity on cancer cells [15], which was extensively studied on different experimental systems, making it a candidate for a cancer chemotherapeutic agent [16]. Previously, we have shown that ART is a powerful inducer of reactive oxygen species (ROS) in tumor cells, triggering DNA damage including 8-oxo-guanine and DSB [17, 18]. Experiments with tumor cells other than GBM indicated a link to autophagy, but the precise mechanism of action of ART remained undissolved [19]. Here, we studied the impact of ART as a modulator of TMZ-induced death in glioma cells. We show that established glioblastoma and glioblastoma stem-like cells are sensitized to TMZ by ART co-treatment. The underlying mechanism does not rest on amelioration of DNA damage such as DSB, but includes ART-mediated inhibition of senescence, which is efficiently triggered by TMZ. The therapeutic effect of TMZ was also found being enhanced in a xenograft mouse model when TMZ was co-administered with ART, which was non-toxic and well tolerated. These pre-clinical data provide a rational basis for a treatment strategy using TMZ in combination with ART, which warrants clinical trials.

## RESULTS

### ART induces apoptosis and necrosis in glioma cells

**Figure 1:**
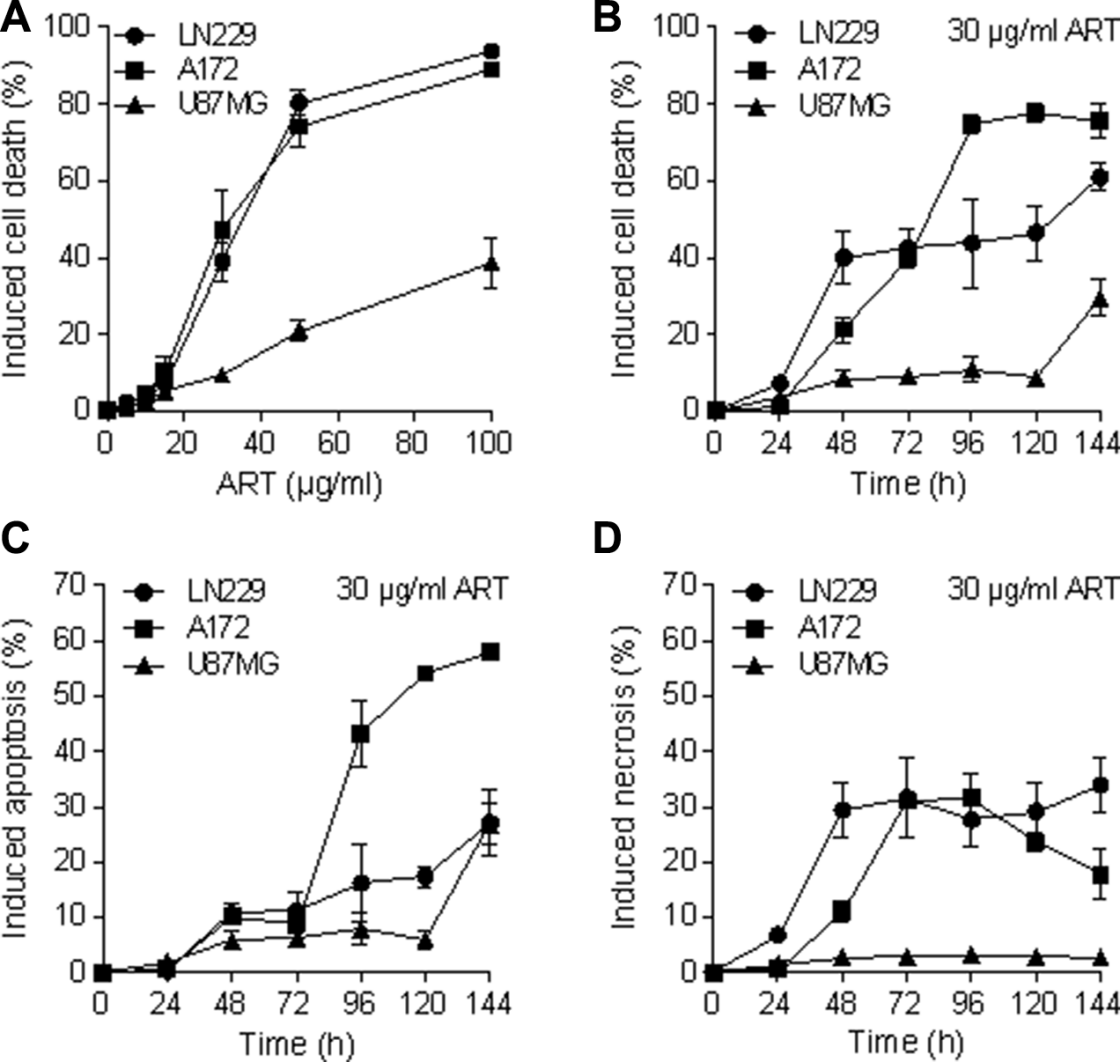
Apoptosis, necrosis and total induced cell death (apoptosis plus necrosis) determined by flow cytometry of annexin V/ PI double-stained glioblastoma cells (LN229, A172, U87MG) after treatment with ART (**A**) Induced cell death measured 72 h after the addition of ART to the medium of exponentially growing cells as a function of dose of ART. The basal levels were subtracted. (**B**) Induced cell death after treatment with 30 μg/ml ART as a function of time following addition of ART to the medium. (**C**) Induced apoptosis following treatment with 30 μg/ml ART as a function of post-exposure time. (**D**) Induced necrosis following treatment with 30 μg/ml ART as a function of post-exposure time. All data are the mean +/− SD of at least three independent experiments.

We used the cell lines LN229, A172 and U87MG, derived from high-grade gliomas, which are p53 wild-type, similar to most of the gliomas [20, 21]. To assess whether ART induces apoptosis and necrosis in glioma cells, they were treated with ART (30 μg/ml). The induction of cell death (sum of apoptosis and necrosis) at different times after the onset of treatment was determined by annexin V/PI staining. As shown in Figure 1A, ART induces cell death dose-dependently, with LN229 and A172 strongly responding, while U87MG was more refractory. The time course experiment shows that cell death occurs 48 h after the onset of treatment and increases further in LN229 and A172 (Figure 1B). Cell death induced by ART was a result of apoptosis (Figure 1C) and necrosis (Figure 1D).

### ART induces ROS and necroptosis

**Figure 2:**
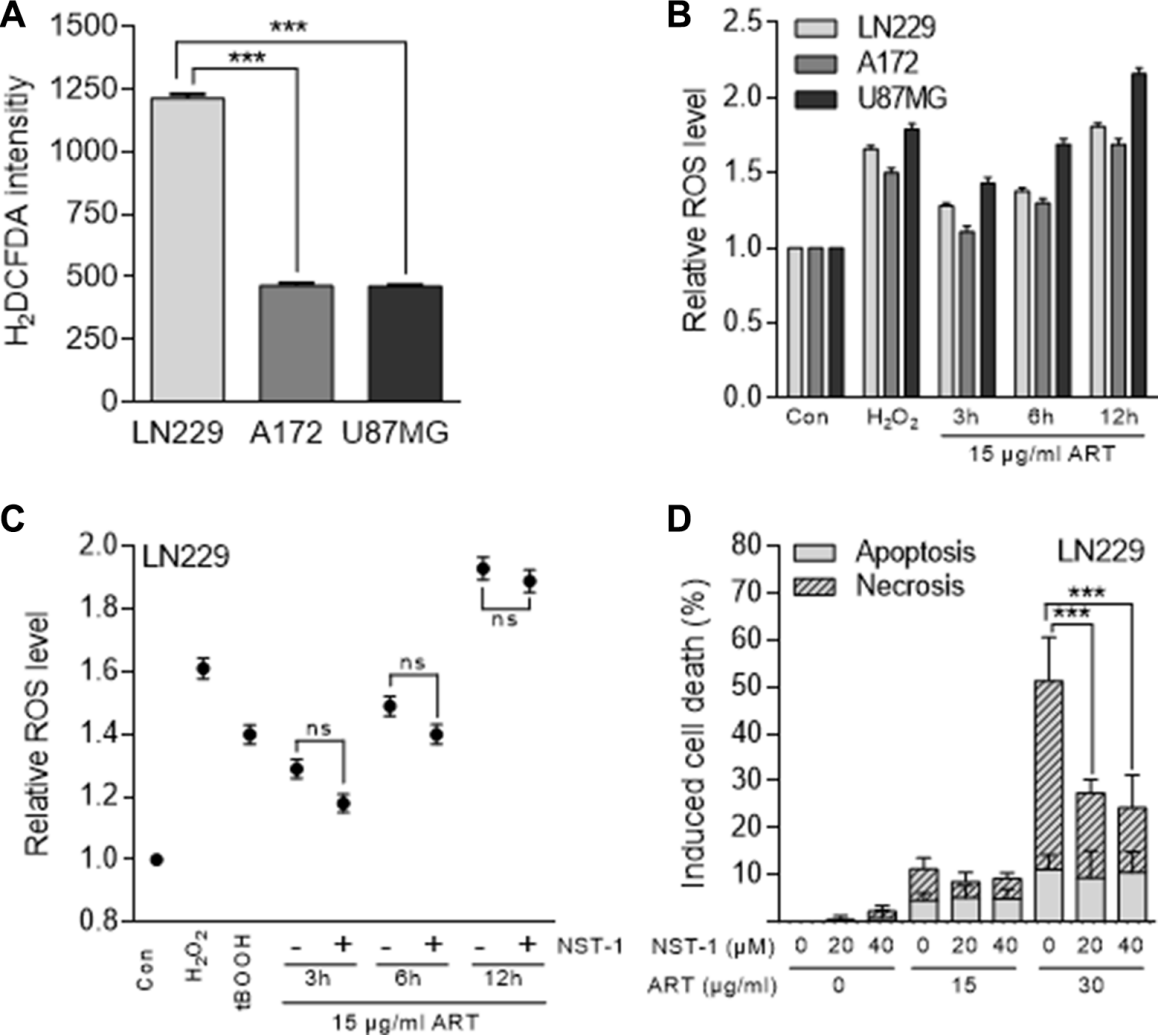
ROS formation measured with the H_2_DCFDA assay and cell death induction in glioblastoma cells (LN229, A172, U87MG) following treatment with ART (**A**) Basal ROS level of untreated glioblastoma cells. (**B**) Relative intracellular ROS level after treatment with 15 μg/ml ART normalized to untreated control cells. Hydrogen peroxide served as positive control. (**C**) Relative ROS level in LN229 cells after treatment with 15 μg/ml ART normalized to untreated control cells in the presence and absence of 20 μM NST-1. As positive controls, cells were treated with hydrogen peroxide (0.5 mM for 1 h) or tBOOH (0.5 mM for 10 min). (**D**) Induction of apoptosis and necrosis were analyzed by the annexin V/PI assay in LN229 cells 72 h after the onset of treatment with ART in the presence and absence of the RIP1 inhibitor NST-1. All data are the mean +/− SD of at least three independent experiments.

As previously shown, ART provokes intracellular radical formation and DNA breaks [18]. Based on this, we hypothesized that ART-induced reactive oxygen species (ROS) are involved in triggering cell death. To determine the basal cellular ROS level, a live cell ROS indicator (H_2_DCFDA) was used. As shown in Figure 2A, A172 and U87MG cells had a similar basal ROS level, which was however significantly lower than that determined in LN229 cells. The induced ROS level after ART treatment increased with time and induction was almost the same in A172 and LN229, and clearly higher in U87MG cells (Figure 2B). This was unexpected since U87MG is the most ART resistant cell line. Obviously, the ART-induced ROS level does not determine the survival response of the cells. Further, we investigated in LN229 cells whether ART induces necroptosis and whether ROS formation is mediated by the necrosome, a trigger of necroptosis. To this end, we used NST-1, which is a specific inhibitor of the receptor-interacting protein 1 (RIP1) that is part of the necrosome. It delivers pro-necroptotic signals including ROS, lysosomal membrane permeabilisation (LMP) and ATP depletion [22]. First, we determined whether NST-1 has an effect on the formation of intracellular ROS induced by ART. As determined 3, 6 and 12 h after the addition of ART to the medium, the treatment with NST-1 did not reduce significantly the intracellular ROS level (Figure 2C). In this experiment, treatment with H_2_O_2_ and tBOOH served as positive controls for intracellular ROS. These data indicate that the necrosome does not contribute to ART-induced ROS production, which is in accordance with the described autocatalytic ROS forming mechanism of ART [23]. Interestingly, if NST-1 was co-administered we observed upon ART treatment a significant reduction of cell death by necrosis (from more than 50% to less than 30%), but not apoptosis (Figure 2D). Since NST-1 is an inhibitor of necroptosis, the data indicate that a significant fraction of cell death induced by ART (administered at high dose level of 30 μg/ml) is due to necroptosis.

### ART ameliorates cell death induced by TMZ

**Figure 3:**
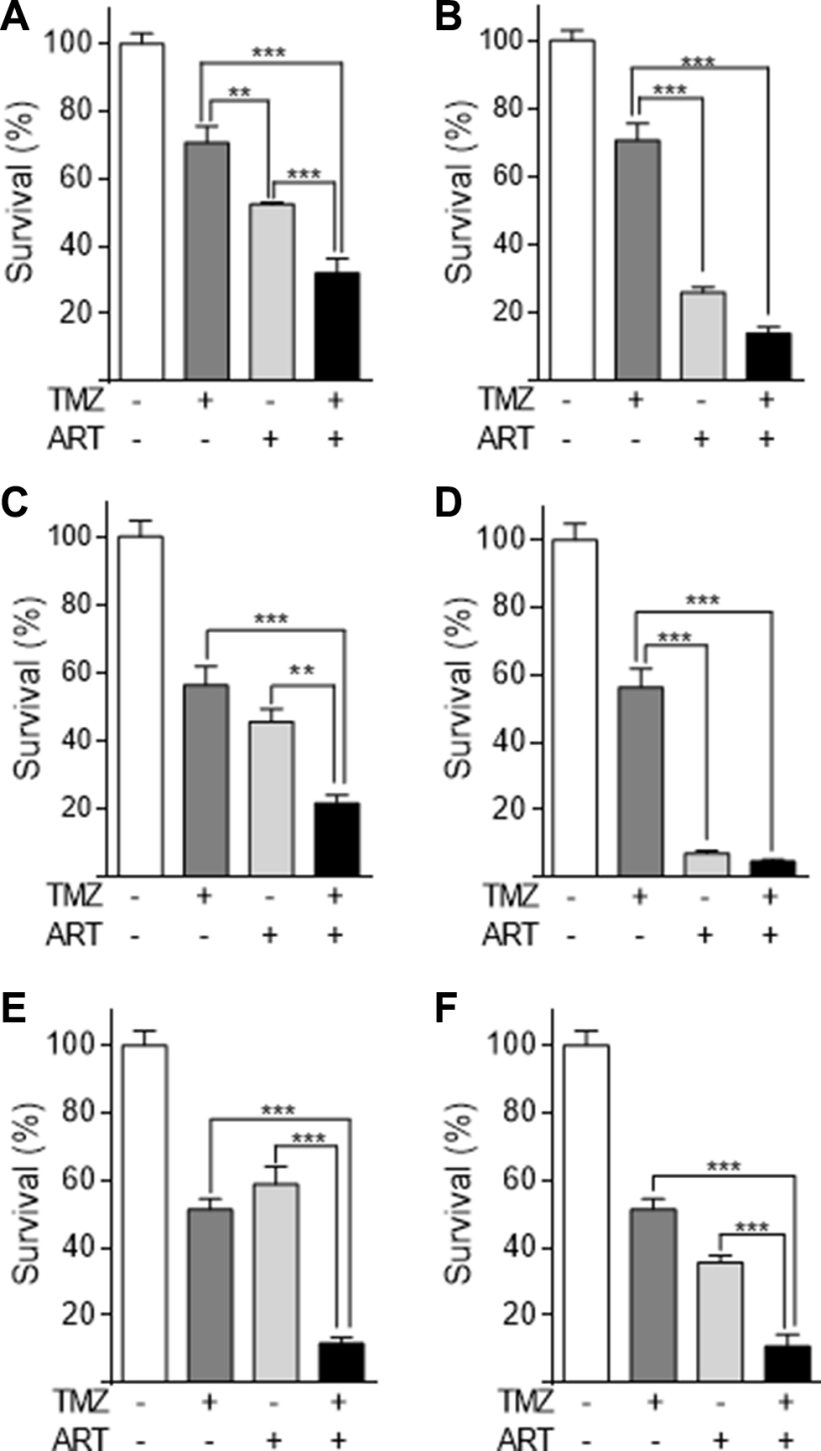
Colony formation assay of glioblastoma cell lines LN229, A172, U87MG treated with TMZ, ART or combined treatment (**A**) LN229 cells were treated with 2.5 μM TMZ and/or 3.8 μg/ml ART. (**B**) The same experiment using 7.5 μg/ml ART. (**C**) A172 cells were treated with 2.5 μM TMZ and/or 3.8 μg/ml ART. (**D**) The same experiment using 7.5 μg/ml ART. (**E**) U87MG cells were treated with 10 μM TMZ and/or 3.8 μg/ml ART. (**F**) The same experiment with 7.5 μg/ml ART. All data are the mean +/− SD of at least three independent experiments.

**Figure 4:**
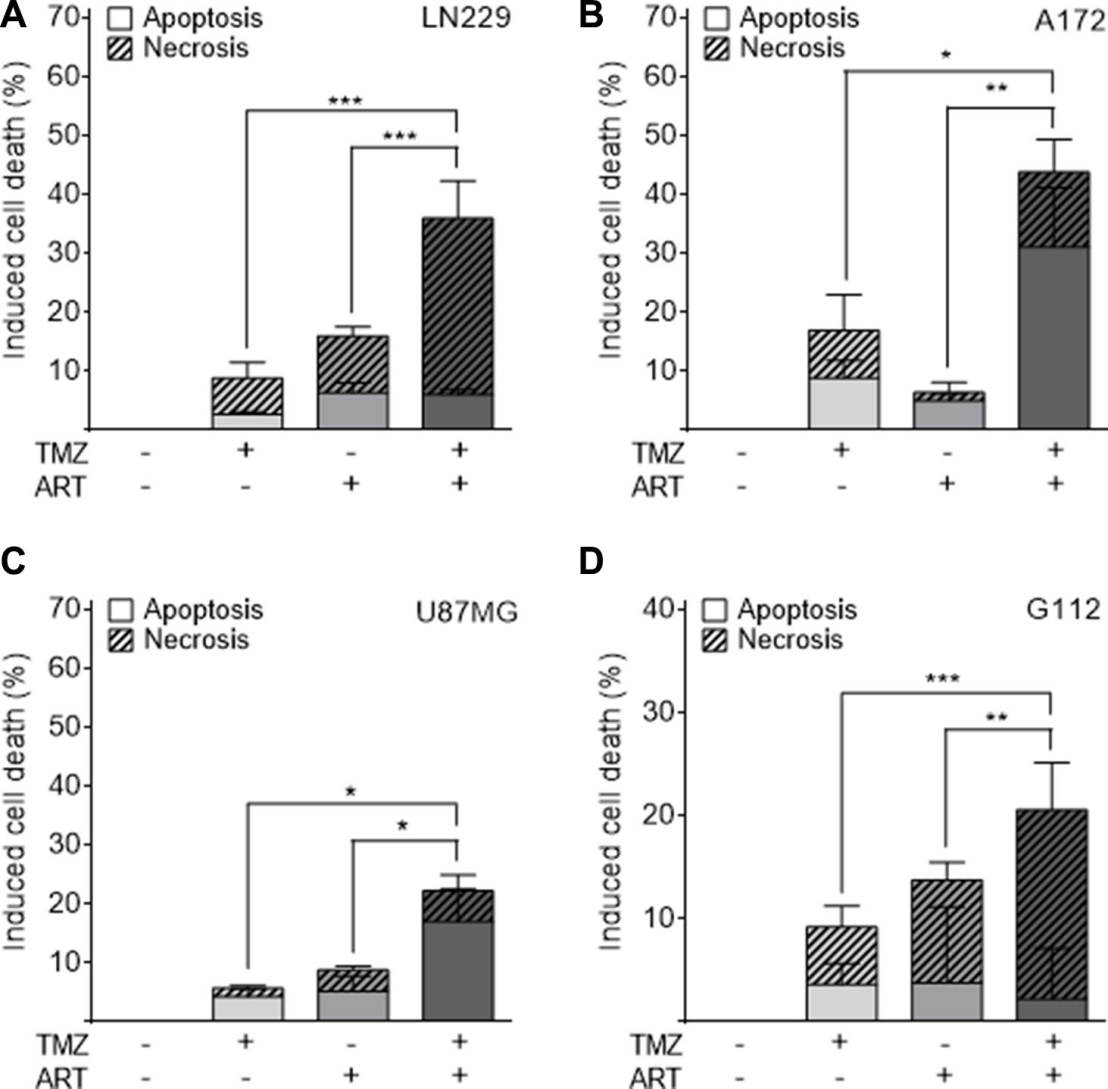
Apoptosis and necrosis induction determined by flow cytometry of annexin V/PI double-stained glioblastoma and glioblastoma stem-like cells Treatment occurred with 50 μM TMZ and 15 μg/ml ART; for G112SP stem-like cells 2 μM TMZ was used. Cells were analyzed 144 h after the onset of TMZ treatment. ART was given to the medium 72 h after TMZ treatment of (**A**) LN229, (**B**) A172, (**C**) U87MG and (**D**) G112SP cells. All data are the mean +/− SD of at least three independent experiments.

Glioblastoma cells respond to TMZ by undergoing apoptosis at late time intervals after TMZ treatment (72 h and later) [9]. The late response is explained by processing of *O*^6^-MeG adducts, which needs mismatch repair and DNA replication, occurring in the 2^nd^ DNA replication cycle after treatment and even later [7]. To investigate whether ART enhances the killing response of glioblastoma cells to TMZ, we compared their survival after single and combined TMZ/ART treatment. First, we conducted colony formation experiments. As shown in Figure 3, ART significantly enhanced reproductive cell death induced by TMZ in LN229 (Figure 3A, 3B), A172 (Figure 3C, 3D) and U87MG cells (Figure 3E, 3F). Similar experiments were conducted by means of annexin V/PI double staining and flow cytometry. ART enhanced cell death (sum of apoptosis and necrosis) of LN229 cells 144 h after TMZ treatment significantly from 16 to 36% (Figure 4A). The same was observed in A172 (Figure 4B) and U87MG cells (Figure 4C). Of note, U87MG were more resistant to TMZ than LN229 and A172 cells, which is conform to data obtained with ART (see Figure 1A).

We also investigated glioblastoma stem-like cells for cell death after combination treatment with TMZ and ART, using the glioblastoma stem-like cell line G112SP. Similar to the glioblastoma lines, G112SP cells were significantly sensitized to TMZ by ART (Figure 4D). We should note that the TMZ concentration in these co-treatment experiments was quite low (50 μM for the glioblastoma lines and 2 μM for G112SP cells), which induced < 20% cell death in each of the lines. The concentration of ART was also low (15 μg/ml); it induced only marginally cytotoxicity (Figure 1). Despite this low dose level of ART, it was clearly effective in ameliorating the TMZ-induced death in glioblastoma cells and stem-like cells.

### Effect of ART on DSB induced by TMZ

**Figure 5:**
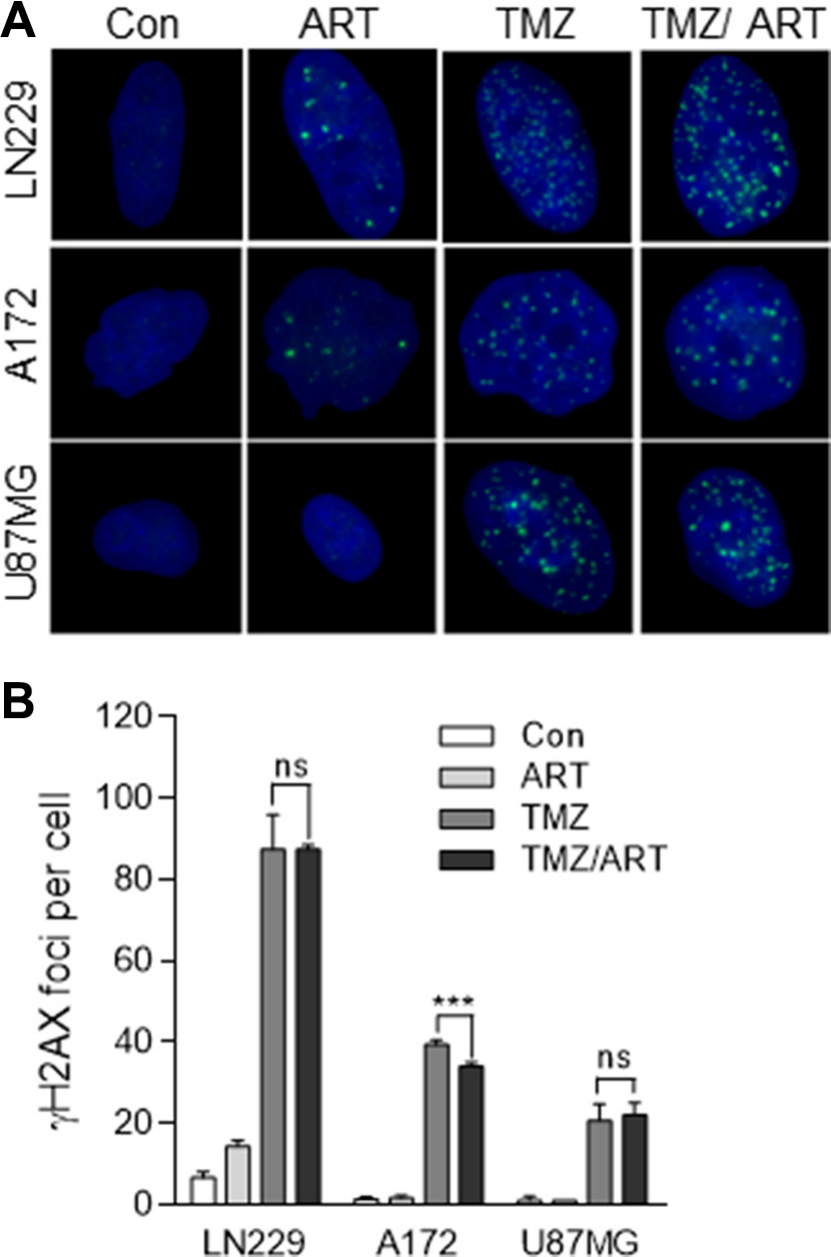
DSB formation in glioblastoma cells (LN229, A172, U87MG) following TMZ and ART Cells were exposed to single or combined treatment with 50 μM TMZ and 15 μg/ml ART. Analysis was performed 96 h after TMZ treatment. Addition of ART occurred 72 h after TMZ treatment. γH2AX foci formation was analyzed by immunofluorescence. (**A**) Representative images of stained nuclei. γH2AX foci are stained in green. Nuclei are counterstained with DAPI in blue. (**B**) Mean number of foci per cell. In each experiment foci in at least 50 nuclei per treatment were determined. Con, untreated control. All data are the mean +/− SD of at least three independent experiments.

In previous studies we showed that both TMZ and ART are able to induce DSB [7, 18]. Although the mechanism for DSB induction for both agents is different, we anticipated that the combination of both treatments causes a synergistic effect. To prove this, we measured DSB after single and combined treatments in glioblastoma cells using immunofluorescence staining for γH2AX. Representative images of immunofluorescent stained nuclei for LN229, A172 and U87MG cells are shown in Figure 5A. The quantification of γH2AX foci revealed that the low dose ART (15 μg/ml) used in this setting did not induce much γH2AX (16, 3 and 2 foci/cell for LN229, A172 and U87MG respectively, which was only for LN229 slightly above the control level). In contrast, TMZ (50 μM) induced a high level of γH2AX foci, which was significant above the control (Figure 5B). Importantly, we did not observe an augmentation of γH2AX foci induced by TMZ in the co-treatment setting (Figure 5B). Obviously, the low dose of ART, which ameliorated TMZ-induced cell death, had no clear impact on the DSB level induced by TMZ.

### ART inhibits Rad51 expression and homologous recombination

**Figure 6:**
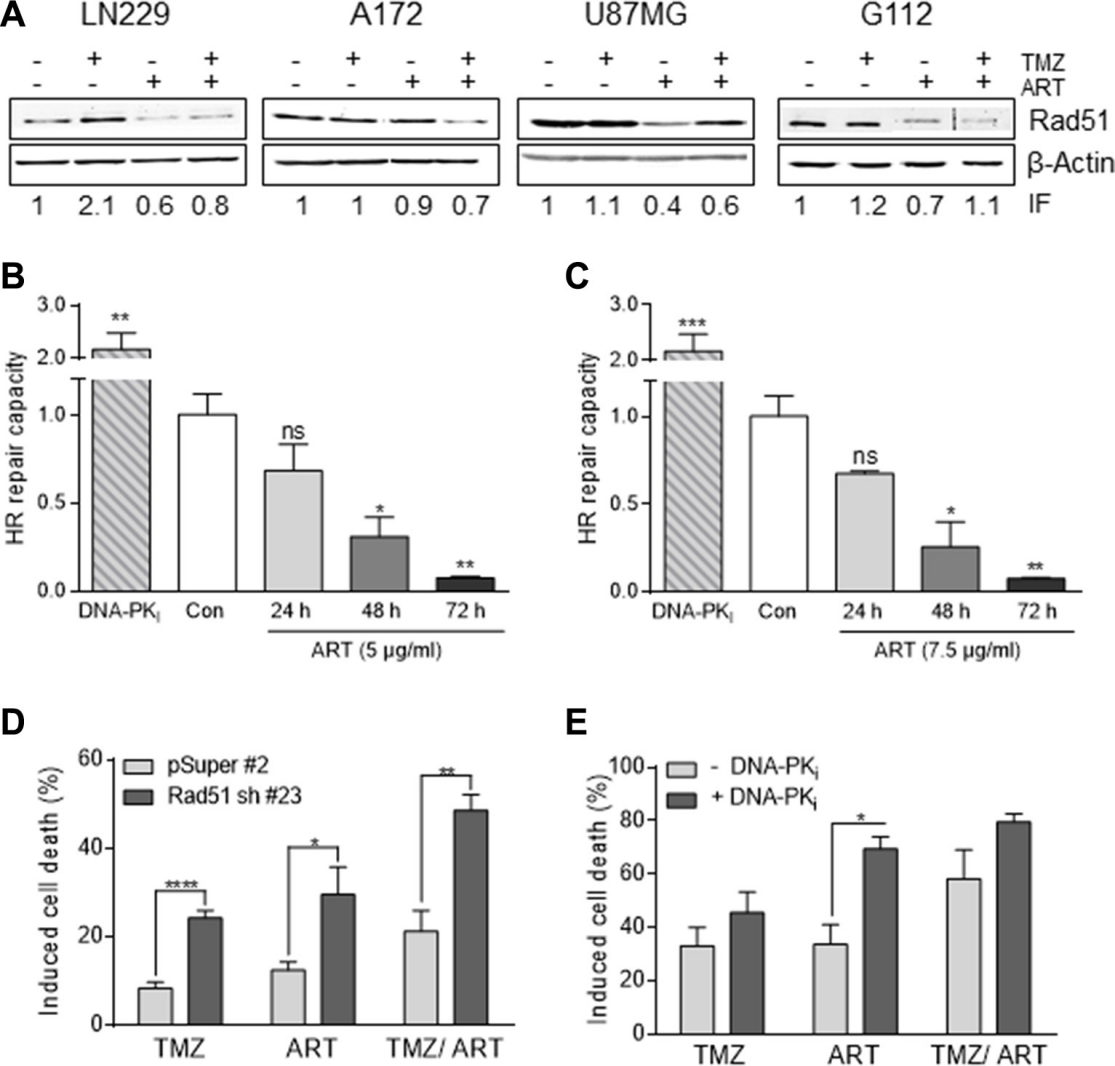
Impact of ART and/or combined treatment on HR/NHEJ in glioblastoma cells (**A**) Western blot analysis of RAD51 expression in LN229, A172, U87MG and G112SP cells. Cells were harvested 96 h following treatment with 50 μM TMZ, or 2 μM TMZ for G112SP cells and/or 15 μg/ml ART. ART was added 72 h after TMZ treatment. β-Actin was used as loading control. The induction factor (IF) was calculated relative to the untreated control. (**B**) and (**C**) HR-capacity of LN229 cells treated with 5 μg/ml (B) or 7.5 μg/ml (C) ART. The DNA-PK inhibitor (DNA-PKi) KU0060648 was used as positive control. (**D**) Induced cell death in LN229 cells transfected with the vector only (pSuper#2) or Rad51 sh vector (Rad51sh clone#23). Cell death (apoptosis plus necrosis) was measured by annexin V/PI double-staining 144 h after the onset of treatment. Cells were treated with 10 μM TMZ and/or 15 μg/ml ART. ART was added 72 h after TMZ treatment. (**E**) Induced cell death in LN229 cells upon pharmacological inhibition of NHEJ by the DNA-PKi. After pre-treatment with KU0060648 (1 μM), cells were treated with 50 μM TMZ and/or 15 μg/ml ART. ART was added 72 h after TMZ treatment, cell death was measured by annexin V/PI double-staining 144 h after TMZ treatment in exponentially growing cell populations. All data are the mean +/− SD of at least three independent experiments.

A mechanism that protects against TMZ-induced cell death is homologous recombination (HR), which was shown to reduce the TMZ-induced DSB level [24]. Therefore, we wondered whether ART might have an impact on the HR efficiency following TMZ administration. A key player in HR is Rad51 [25]. As shown in Figure 6A, Rad 51 expression was enhanced in LN229 cells if they were treated with TMZ. In the presence of ART, however, this enhancement was not observed; there was rather a decline in Rad51 expression. In A172 and U87MG cells we did not observe an enhancement in the Rad51 level following TMZ, however, there was a clear decline in Rad51 protein when cells were exposed to ART. This was also observed in the glioma stem-like cells G112SP (Figure 6A). Additionally, we determined the HR capacity using an HR plasmid assay (as described in Material and Methods). As a control, we treated the LN229 cells with an inhibitor of DNA-PK, which blocks non-homologous end-joining (NHEJ) and at the same time ameliorates the HR level, which is known to be a compensatory effect [24]. Treatment with ART inhibited the cellular HR activity time dependently, with a nearly complete inhibition achieved 72 h after addition of ART to the cells. In these experiments we used low non-toxic doses of ART of 5 μg/ml (Figure 6B) and 7.5 μg/ml (Figure 6C) to avoid any interference with killing effects.

### Effect of HR on TMZ/ART-induced cell death

Does downregulation of HR impact survival following TMZ and ART? This question was addressed by downregulating Rad51 with shRNA. As shown in Figure 6D, Rad51 downregulation results in an enhancement of cell death (apoptosis plus necrosis measured by annexin V/PI) following treatment with TMZ and also with ART. In the combined setting TMZ/ART cell death was also significantly enhanced in Rad51 downregulated cells (Figure 6D). This supports the critical role of HR in defense against TMZ- and ART-induced cell death. Inhibition of NHEJ by DNA-PK_i_ was without effect on TMZ-induced cell death while ameliorating death following ART (Figure 6E). This is in line with previous data showing a marginal role for NHEJ in TMZ induced death of glioma cells [26], while for ART both HR and NHEJ are involved in the defense against DNA damage [17, 18]. Overall, the data support the notion that HR has a significant impact on TMZ/ART induced cell death.

### Effect of ART on TMZ-induced autophagy

**Figure 7:**
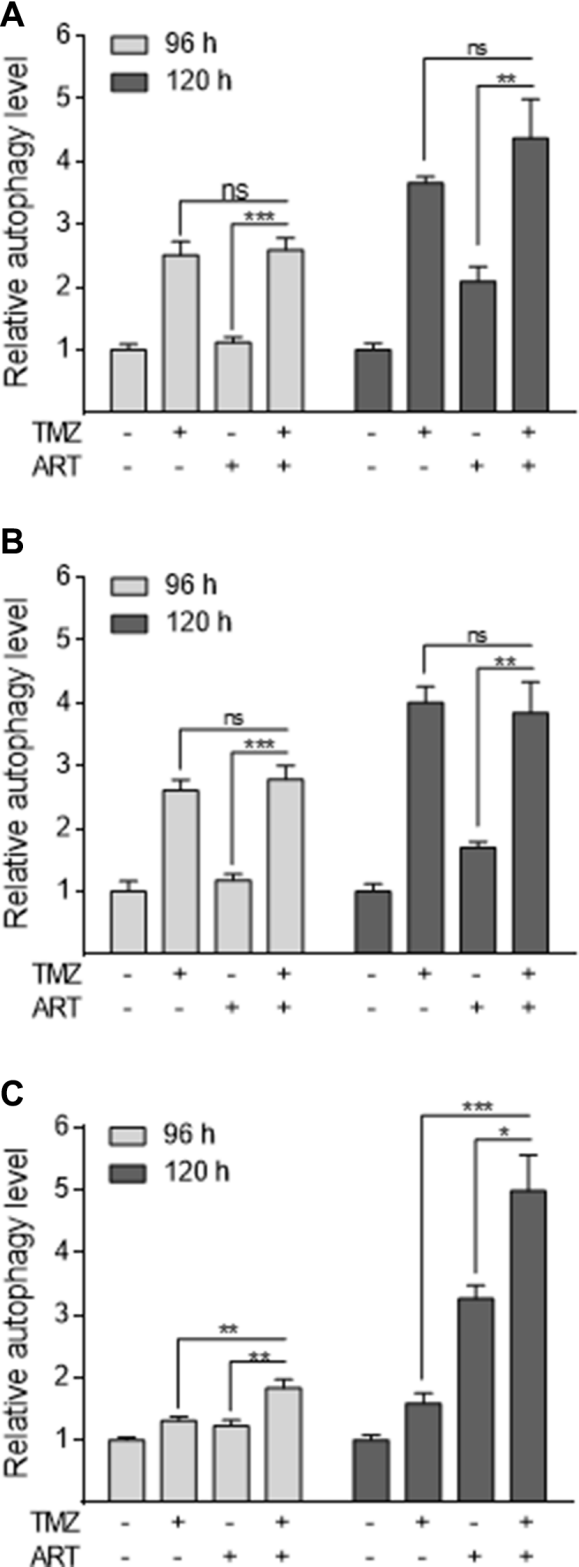
Induction of autophagy in glioblastoma cells measured by the CytoID assay The extent of autophagy is normalized to the untreated control. LN229 (**A**), A172 (**B**) and U87MG (**C**) cells were treated with 50 μM TMZ and/or 15 μg/ml ART, administered 72 h after the begin of TMZ treatment. Autophagy was measured 96 and 120 h after the onset of treatment. All data are the mean +/− SD of at least three independent experiments.

We have recently demonstrated that TMZ induces autophagy, which serves as a survival mechanism inhibiting apoptosis [11]. It was also shown that ART interrupts the autophagy process in MCF-7 breast cancer cells [19]. Therefore, we hypothesized that a possible mechanism of ART for sensitizing glioblastoma cells to TMZ rests on affecting the autophagy process. We analyzed glioblastoma cells by staining autophagic vesicles (Cyto ID assay) after treatment with TMZ. TMZ clearly induced autophagy in LN229 glioblastoma cells, while treatment with ART did not affect significantly the basal autophagy level in LN229 and A172 cells, and only slightly enhanced the level in U87MG cells (Figure 7). Combination of ART and TMZ did not show a reduction of autophagy compared to TMZ single treatment demonstrating that ART, administered in this low dose range (15 μg/ml), does not stimulate TMZ-induced apoptosis by suppression of autophagy.

### Effect of ART on TMZ-induced senescence

**Figure 8:**
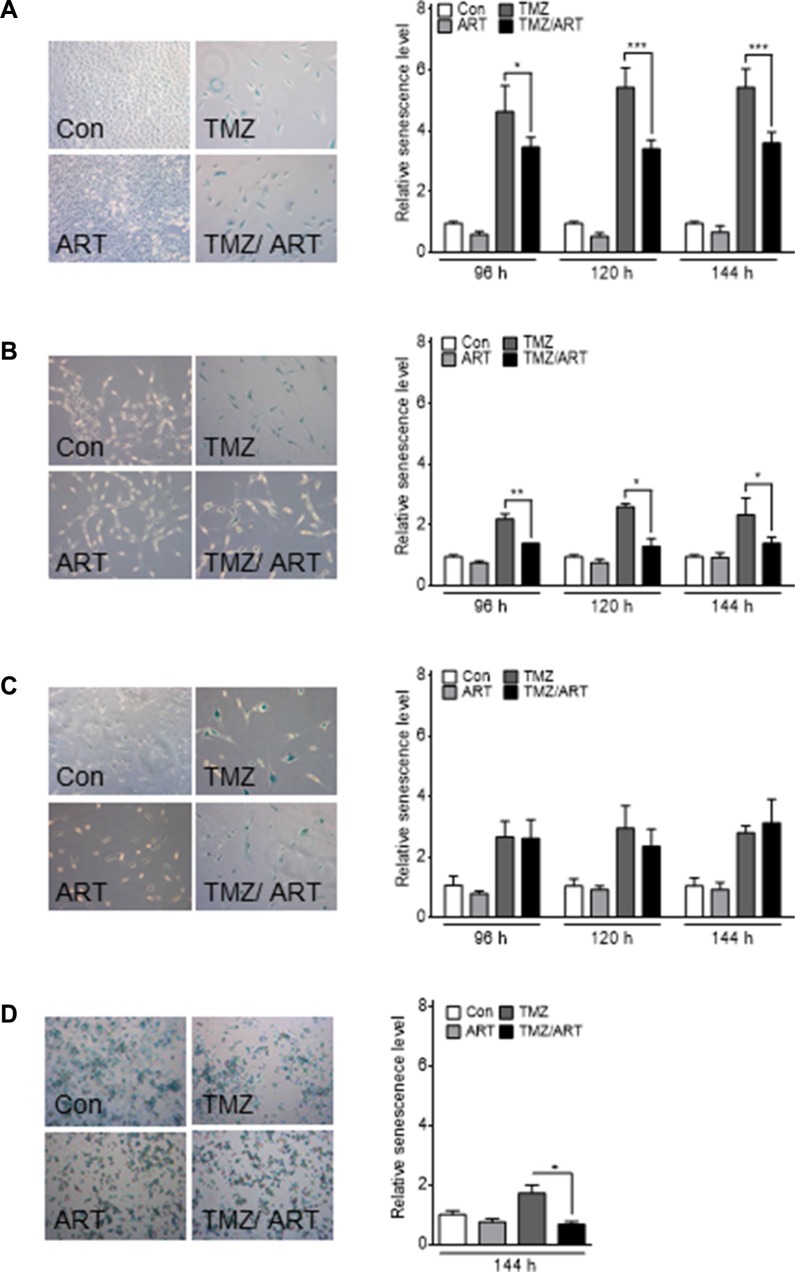
Induction of senescence analyzed by the C_12_FDG assay in glioblastoma cell lines Cells were treated with 50 μM TMZ and/or 15 μg/ml ART. For G112SP stem-like cells 2 μM TMZ was used. Analysis was performed 96, 120 and 144 h after the onset of treatment. Treatment with ART occurred 72 h after the onset of TMZ treatment. The senescence level was normalized to the untreated control. Representative pictures of SA-β-gal staining are shown in the left panels for (**A**) LN229, (**B**) A172, (**C**) U87MG and (**D**) G112SP cells. All data are the mean +/− SD of three independent experiments.

In previous studies we showed that TMZ induces senescence, which reduces the level of cell death by apoptosis and necrosis [11]. This let us to consider the idea that ART might have an impact on TMZ-induced senescence. We investigated the induction of senescence in glioblastoma cells using the C_12_FDG assay, which measures senescence-associated β-galactosidase (SA-β-gal) activity [27]. Treatment with low dose of ART does not lead to induction of senescence in LN229, A172 and U87MG cells (Figure 8A–8C). In contrast, TMZ (50 μM) induced a high level of senescence in LN229 cells (Figure 8A) and a moderate level in A172 and U87MG cells (Figure 8B, 8C). TMZ-induced senescence was significantly reduced in LN229 and A172 cells when they where post-treated with ART (Figure 8A, 8B). The generally more resistant U87MG cells were not responding (Figure 8C). TMZ-induced senescence was also reduced by ART treatment in glioblastoma stem-like cells (Figure 8D). The data support the notion that ART has a potential to impact TMZ-induced senescence.

### ART enhances the therapeutic effect of TMZ

**Figure 9:**
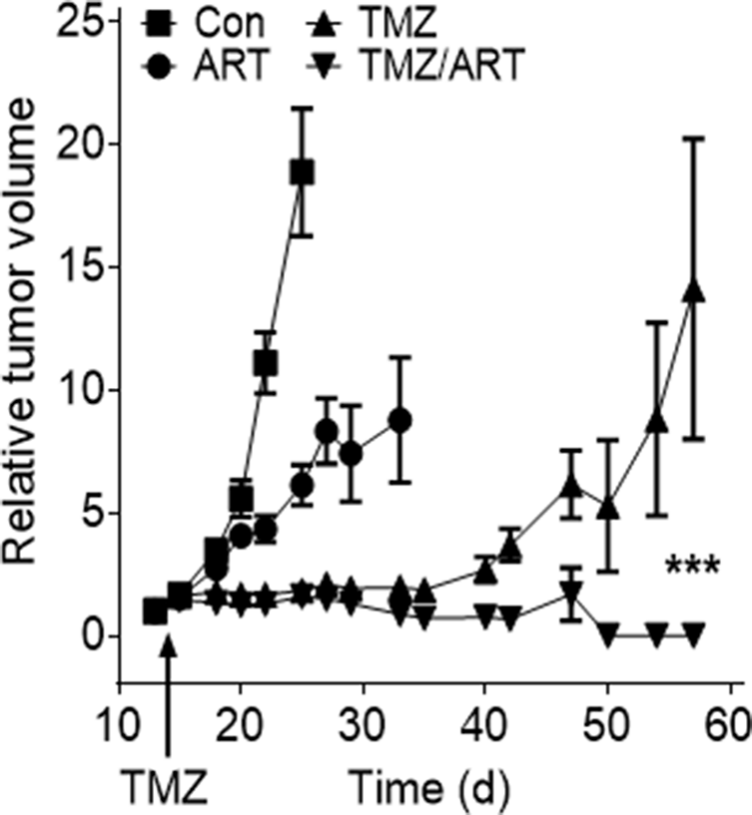
Tumor growth after subcutaneous implantation of U87MGΔ cells in nude mice non-treated and treated with TMZ, ART and TMZ (single dose) followed by repeated doses of ART Tumor volume was set in relation to the tumor size in a given individual determined one day before TMZ injection (200 mg/kg body weight). ART (1 mg/ml) was given with the drinking water, beginning one day after TMZ injection.

Having shown that ART enhances the killing effect of TMZ *in vitro*, we studied whether ART has an impact on tumor growth following TMZ in a mouse model. We used the line U87MGΔ because of a reasonable take on rate if transplanted subcutaneously into nude mice. Upon appearance of tumors, mice were treated with a single dose of TMZ (200 mg/ kg body weight) followed by treatment with ART, which was administered daily in drinking water (1 mg/ ml). As shown in Figure 9, ART and TMZ as single treatment caused a reduction in tumor growth, with TMZ being more effective. The combined treatment, however, inhibited tumor growth most efficiently with a complete remission of tumors in the observation period. Furthermore, mouse survival was investigated in an orthotopic tumor model, in which LN229 cells were transplanted intracranially in nude mice. The mean survival time in the control and ART group was 37 and 40 days, respectively. Repeated low dose TMZ treatment enhanced the mean survival period to 169 days while concomitant TMZ and ART treatment resulted in a clearly extended mean survival period of 242 days. The treatments with TMZ and ART over a long period were well tolerated as revealed by the body weight of mice (Supplementary Figure S1). The data obtained with the glioblastoma xenograft model support the notion that TMZ followed by ART or administrated in combination with ART ameliorates the curative effect of TMZ.

## DISCUSSION

ART, a semi-synthetic derivative of the active ingredient of extracts of *Artemisia annua* L., which are used for centuries in TCM for the treatment of various inflammation-associated diseases [16], has come to attention in conventional medicine because of its antibiotic effect on plasmodia [28]. This made it a first-line drug for the therapy of malaria, since plasmodia developed resistance to conventional drugs such as chloroquine. The mechanism of death of plasmodia is thought to result from iron-stimulated ROS production by ART in plasmodia host cells, the erythrocytes, and it has been concluded that membrane damage kills the microorganism in the host cells [29, 30]. Later on it has been shown that ART is also able to kill mammalian cells, including cancer cells [31]. The mechanism of cancer cell death has been studied extensively and it has been shown that ART generates ROS, which attacks DNA resulting in oxidative DNA damage that finally leads to DSB formation and induction of apoptotic and necrotic cell death [17, 18]. Since cells defective in DSB repair are hypersensitive to ART, it was concluded that in mammalian cells DSB are the trigger for ART-induced cell death [17]. ART exerts remarkable killing effects in LN229 glioblastoma cells, which was preceded by a continuous wave of ROS and DSB formation [18]. Since TMZ also produces DSB following processing of *O*^6^-MeG/T mispairs by the mismatch repair machinery [9], we hypothesized that the killing effect of TMZ could be ameliorated by concomitant ART treatment.

Here we show for glioblastoma and glioblastoma stem-like cells that ART post-treatment enhances the killing effect of TMZ. This was shown for reproductive cell death and the end-points apoptosis and necrosis. TMZ induces mostly apoptosis in glioblastoma cells [26, 32], which is a late response [26], and this response was enhanced when ART was administered up to 72 h after TMZ application. Simultaneous treatment with TMZ and ART was ineffective in our *in vitro* setting, especially if high doses of ART (> 30 μg/ml; > 40 μM) were used (data not shown). This is presumably due to the proliferation-inhibiting effect of ART, which is obvious at toxic doses and interferes with the processing of TMZ-induced *O*^6^-MeG lesions that needs replication, which is mandatory for TMZ-induced apoptosis [26].

It is also important to note that TMZ was used at a moderate dose not exceeding 50 μM. For comparison, the typical plasma level of TMZ achieved in cancer therapy is 15–30 μM [33–35]. As noted above, the concentration of ART used in our co-treatment experiments was also low (< 30 μg/ml, i.e. < 40 μM. This low dose level did not induce significant DSB and apoptosis/necrosis when administered as single compound. For comparison, the plasma ART level in malaria therapy is in the range of 0.4–0.6 μg/ml (corresponding to 1–1.5 μM) when the drug is administered as infusion with 1–8 mg/kg body weight of the patient [36, 37]. Thus, for concomitant administration of TMZ with ART in glioma therapy higher ART doses are presumably required, which may still be tolerated [38].

Interestingly, in the low ART dose range used we did not observe an increase in DNA damage following TMZ/ ART, compared to TMZ single treatment. Thus, the amount of DSB, quantified as γH2AX foci, remained the same. This is likely due to the low concentration of ART we have chosen in the combined experimental setting. Since the dose of ART applied in the combined treatments was below the effective level of DSB formation, we hypothesized another other mechanisms being involved in TMZ sensitization. Previously we have shown that apoptosis induced by TMZ is preceded by autophagy, which is triggered by the same critical DNA damage that induces apoptosis, namely *O*^6^-MeG [11]. Since inhibition of *O*^6^-MeG-triggered autophagy in an experimental setting enhanced the killing effect of TMZ, it was conceivable that ART has an impact on TMZ-induced autophagy, thus blocking a pro-survival mechanism. This supposition prompted us to study the autophagy response of glioblastoma cells following TMZ and ART treatment. We observed that TMZ treatment induces autophagy, confirming previous data. However, the autophagy level was not altered by ART, showing that ART in the dose range used does not inhibit the autophagy process triggered by TMZ. We should note that ART was previously shown to have the potential of inhibiting autophagy [19], which was however not assessed in the context of TMZ and low doses of ART.

Previously, we also showed that the *O*^6^-MeG lesion activates senescence, which is like autophagy a survival mechanism [11]. Here we confirm that TMZ induces senescence in glioblastoma cells. Strikingly, the induction of senescence was significantly reduced when ART was administered after TMZ application. Therefore, we conclude that ART-induced abrogation of TMZ-induced senescence is causally involved in enhancing the killing effect of TMZ. The mechanism of senescence triggered by TMZ is not entirely known and, likewise, the mechanism of ART that attenuates senescence induction. For UV irradiation it was shown that concomitant activation of p53 and AKT can trigger cellular senescence [39]. Since the AKT pathway is inhibited by ART [40, 41], the ART provoked inhibition of TMZ-induced senescence (as observed in our experiments) might be caused by inactivation of players involved in the AKT pathway. Clearly more molecular studies using the combined treatment setting are required to solve this intriguing question.

The critical TMZ-induced DNA lesion triggering cell death in a clinically relevant dose range is *O*^6^-MeG. This damage causes mispairings with thymine, which are processed by mismatch repair, resulting in replication blocking secondary lesions, very likely extended DNA gaps [42] and DSB in the second post-treatment replication cycle [7]. These secondary replication blocking lesions are processed or tolerated by HR [43], which has a strong impact on the survival of cells following DNA methylation [24]. One of the key players of HR is Rad51, whose downregulation significantly sensitized glioblastoma cells to TMZ [25]. This background data prompted us to address the question whether ART impacts the process of HR following TMZ treatment. Here we show that ART downregulates the level of Rad51 protein, as shown by western blot experiments, and the HR frequency, using a functional assay. We therefore conclude that Rad51 downregulation together with inhibition of replicative senescence contributes to the enhancing effect of ART on TMZ-induced cell death. The data are in line with a recent study showing that ART causes a reduction in the Rad51 level in ovarian cancer cells thus sensitizing them to cisplatin [44].

In the mouse xenograft experiments we observed a reduction in tumor growth when mice were treated with a single dose of TMZ followed by repeated treatments with ART. Some tumors completely disappeared and others melted down to a scar tissue. Additionally, repeated treatment with TMZ in combination with ART led to an increased survival in a mouse brain tumor model. Therefore, it is likely that repeated treatments with TMZ together with ART are superior to single TMZ treatment, as it was applied in the experimental *in vitro* setting shown here. Of note, in the clinical setting patients receive TMZ daily (serum half-life ~2 h) [34], i.e. they are chronically and pulsatile exposed to the alkylating agent, which is well tolerable even after long-term treatment [45]. If chronic TMZ exposure would occur together with ART, the processes we have studied *in vitro* are expected to go on repeatedly in the cancer cells *in vivo*, which might strongly enhance the effectiveness of treatment. Importantly, the side effects of combined treatment on tumor bearing mice were negligible and, overall, the continuous treatment with ART was well tolerated.

ART and its metabolies such as dihydroartemisinin have been studied previously in several co-treatment settings *in vitro* showing an ameliorating effects on the cytotoxicity of carboplatin in ovarian cancer cells [46], doxorubicin in leukemic T cells [47], gemcitabine in hepatoma cells [48], cyclophosphamide in lung cancer cells [49], and ionising radiation in lymphoma cells [50, 51]. For TMZ it was shown that ART enhances the killing response of rat C6 glioma cells [52], Here, we extend these studies and report that combined treatment of human glioblastoma cells with TMZ and low dose of ART is superior over single treatments, enhancing killing of glioblastoma and glioblastoma stem-like cells *in vitro* and in a xenograft mouse model. In conclusion, sequential treatment with TMZ and ART appears to be a reasonable strategy for glioblastoma treatment, worth to be proven in clinical trials.

## MATERIALS AND METHODS

### Cell lines and culture conditions

LN229 and A172 glioblastoma cell lines were provided by Dr. Weller (Department of Oncology, University Hospital Zurich). The U87MG glioma cell line was purchased from CLS Heidelberg and U87MGΔ expressing the truncated form of the EGFR were a kind gift of Dr. Cavenee, San Diego, USA [53]. Stable transfected LN229 cells (the clone pSuper #2 for the control vector and clone Rad51 sh #23) were generated in this laboratory as previously described [25]. All cell lines were maintained in Dulbecco's modified Eagle's medium (DMEM) with 10% fetal bovine serum (FBS) at 37°C and 5% CO_2_ atmosphere. The transfected LN229 cells were selected with 0.75 mg/ml G418 (Millipore). Glioma stem-like cells G112SP were isolated from the human glioma cell line G112 [54] by selecting cell populations capable of continuous propagation in serum-free medium. G112SP cells possess stemness attributes including the propensity for self-renewal, phenotypic plasticity and the ability to generate clonal gliomaspheres *in vitro.* They are highly tumorigenic and recapitulate distinct properties of GBM such as invasive tumour growth, intratumoural heterogeneity and radiation resistance [55]. The G112SP cells were grown under serum-free condition in Neurobasal-A medium (Life Technologies) containing B-27 supplement (Life Technologies), 20 ng/ml EGF (Biochrom), 10 ng/ml FGF-basic (Biochrom) and 0.1% bovine serum albumin (BSA). Cells were checked for mycoplasma contamination before experimental use, cultured for a maximum period of 12 weeks and then replaced.

### Drugs and drug treatment

For *in vitro* application TMZ (gift from Schering-Plough) was dissolved in 30.9% dimethyl sulfoxide (DMSO). ART (kindly provided by Dr. Jansen, Dafra Pharma International) was dissolved in 2% DMSO/phosphate buffered saline (PBS). Necrostatin-1 (NST-1, Enzo Life Science) was dissolved in 0.02% DMSO/PBS. O^6^-benzylguanine (O^6^-BG, Sigma-Aldrich) was dissolved in DMSO. All drugs were added to the cell culture medium as single application and maintained within the medium until the end of incubation time. ART treatment was performed 72 h after TMZ application. To inhibit MGMT activity in the G112SP line the cells were pretreated with 10 μM *O^6^*-BG prior TMZ treatment. To inhibit DNA-PK activity cells were incubated with 1 μM KU-0060648 (Selleckchem) 1 h prior TMZ as well as ART treatment. For *in vivo* use, TMZ was dissolved in 16.7% DMSO/0.9% NaCl (subcutaneous transplantation) and 1.4% DMSO/0.9% NaCl (intracranial transplantation). ART was dissolved in 1% ethanol (subcutaneous transplantation) and 50 mM KH_2_PO_4_/5% saccharose (intracranial transplantation).

### Determination of apoptosis and necrosis

The frequency of apoptosis and necrosis was determined by annexin V/propidium iodide (PI) double staining and quantified by flow cytometry with FACS Canto II (Beckton Dickinson) as previously described [56].

### Determination of ROS formation

ROS formation was measured using H_2_DCFDA (Invitrogen). After pretreatment with 4 μmol H_2_DCFDA in serum-free and pH indicator-free medium for 30 min, cells were treated with NST-1 and ART at the indicated time points. After incubation cells were washed twice with PBS and analyzed (FACSCalibur, Becton Dickinson). Relative ROS level was determined by comparing the fluorescence intensity of treated with untreated cells.

### Clonogenic survival

In colony formation assays glioblastoma cells LN229, A172 and U87MG were seeded at a density of 250 cells/ml. They were allowed to attach and 24 h later treated with TMZ and/or ART. After 11–14 days, the colonies were fixed with 100% methanol for 20 min, stained with crystal-violet (1 g/l dH_2_O) for 30 min and colonies containing at least 40 cells were counted and presented graphically as a percentage of untreated cells (control). The experiments were repeated at least three times.

### Immunofluorescence

Cells were seeded on cover slips. After treatment with TMZ and ART the cells were fixed with 4% formaldehyde for 15 min, washed with PBS, permeabilized in ice-cold methanol for 10 min, rehydrated in PBS and blocked with 10% normal goat serum and 0.25% Triton-X100 for 1 h. Cover slips were incubated overnight with a γH2AX-antibody (Millipore), washed with PBS and incubated with Alexa Fluor 488 anti-mouse secondary antibody (Invitrogen) for 1 h. Cover slips were covered with Vectashield mounting media (Vector Labs) containing DAPI and fixed on glass slides using nail varnish. Metafer software was used for automatic scoring of 200 cells per sample and BIC Macro Toolkit software (Bioimaging Center, University Konstanz) for automatic foci quantification.

### Protein extract preparation and western blot analysis

Whole protein extracts were prepared from LN229, A172, U87MG and G112SP cells not treated and treated as described. Cells were trypsinized and washed with PBS, then lysed, sonified and boiled in Laemmli sample buffer (60 mM Tris-Cl (pH 6.8), 2% SDS, 10% glycerol, 5% β-mercaptoethanol, 0.01% bromophenol blue) at 95°C for 5 min. Cell extract was separated on a 12% SDS polyacrylamide gel at 100V and blotted onto a nitrocellulose membrane for 90 min at 300 mA using buffer composed of 25 mmol/ L Tris-HCl, 86 mmol/ L glycin and 20% methanol. Anti-Rad51 (Abcam) and Anti-β-Actin (Santa Cruz) were used for immunodetection. The induction factor (IF) of Rad51 referred to β-actin (loading control) and the non-treated control was set to 1.

### Homologous recombination activity assay

To measure the capacity of cells to repair DSB by HR, LN229 cells were stably transfected with a pDRGFP plasmid (Addgene). The plasmid bears two non-functional *GFP* genes. One truncated, the other containing a recognition site for I-SceI endonuclease. Upon transient transfection with I-SceI expressing plasmid (Addgene), the endonuclease cleaves the modified GFP gene leading to a DSB. If this DSB is getting repaired by HR, using the sequence of the truncated *GFP* gene, a functional GFP protein is generated, which can be quantified by flow cytometry. Cells were analyzed 72 h after transfection with 1 μg pCβASceI using the transfection kit Effectene (Quiagen). During transfection, the cells were incubated in the presence of ART (24 h, 48 h, 72 h). The DNA-PK inhibitor KU0060648 (Selleckchem) was used for comparison at a final concentration of 1 μM. We expected that the inhibition of DNA-PK leads to a moderate increase in HR activity due to the lack of competition by NHEJ for the repair of DSB (Shrivastav, 2008). Cells were trypsinized and washed with PBS and measured by flow cytometry using FACS Canto II (BD Biosciences). Data were analysed with BD FACSDiva™ software.

### Determination of autophagy (CytoID assay)

The flow cytometric investigation of Cyto-ID Green Detection Reagent stained cells was performed according to the manufacturer's protocol (Cyto-ID Autophagy Detection Kit, Enzo Life Sciences). Cells were analyzed using FACSCanto II (Becton Dickinson) as discussed previously [11]. Relative autophagy level was normalized to untreated control cells.

### Determination of senescence

Replicative senescence was determined as described previously [27]. Briefly, cells were preincubated with 100 mM bafilomycin A1 (Sigma) for 1 h, then stained with 33 μM C_12_FDG (Invitrogen) in medium for 60 min before harvest. At the end of experiment (144 h following TMZ treatment), cells were trypsinized and washed in cold PBS. Flow cytometry of the cell suspension in PBS was performed at FACSCanto II (Becton Dickinson). Relative senescence level was normalized to untreated control cells. In addition to FACS measurement, senescence-associated beta-galactosidase (SA-β-gal) activity was detected cytochemically via Senescence β-Galactosidase Staining Kit (CST) according to the manufacturer's protocol. Treated glioblastoma cells and stem-like G112SP cells were fixed and stained for 16 h for β-galactosidase.

### Xenograft mouse experiments and intracranial model

For tumorigenesis experiments, we used U87MGΔ cells [53] for subcutaneous implantation. 5 × 10^6^ cells diluted in 100 μl PBS were injected subcutaneously in the dorsal flanks of BALB/c nude mice (Charles River). After mice developed palpable tumors, a single TMZ dose of 200 mg/kg body weight was injected intraperitoneally. One day after TMZ treatment, ART (1 mg/ml) was given continuously in drinking water supplemented with 2% saccharose. The tumor volume was calculated according to the formula TV = L × W^2^/2 (TV tumor volume; L length; W width). At least 5 animals were used per treatment group. The intracranial model was performed with LN229 cells. 1 × 10^5^ cells in 2 μl PBS were injected into the brain of NMRI nu/nu mice (Charles River). Three weeks after implantation, tumors developed as verified by MRT. At this point low dose TMZ was administered intraperitoneally (5 mg/kg body weight) five times a week for the duration of six weeks. In parallel ART (100 mg/ kg bodyweight) was applied orally five times a week for the duration of nine weeks and afterwards three times a week. Survival of mice was determined over a time of 329 days. Per treatment group at least 7 animals were included. All animal procedures were carried out according to the guidelines of the German regulations for animal welfare. Protocols were approved by the local ethic commission.

### Statistics

All experiments were repeated at least three times and the data were evaluated statistically using the ANOVA test (*p < 0.05; **p < 0.01; ***p < 0.001; ****p < 0.0001)

## SUPPLEMENTARY MATERIAL FIGURE


