# Supplementary material for: Artesunate enhances the therapeutic response of glioma cells to temozolomide by inhibition of homologous recombination and senescence
Source: Oncotarget. 2016 Sep 12;7(41):67235–50. doi: 10.18632/oncotarget.11972 (PMC5341871; doi:10.18632/oncotarget.11972)
Supplement: Supplementary file 1 [file oncotarget-07-67235-s001.pdf]

Artesunate enhances the therapeutic response of glioma cells to temozolomide by inhibition of homologous recombination and senescence

Supplementary Materials

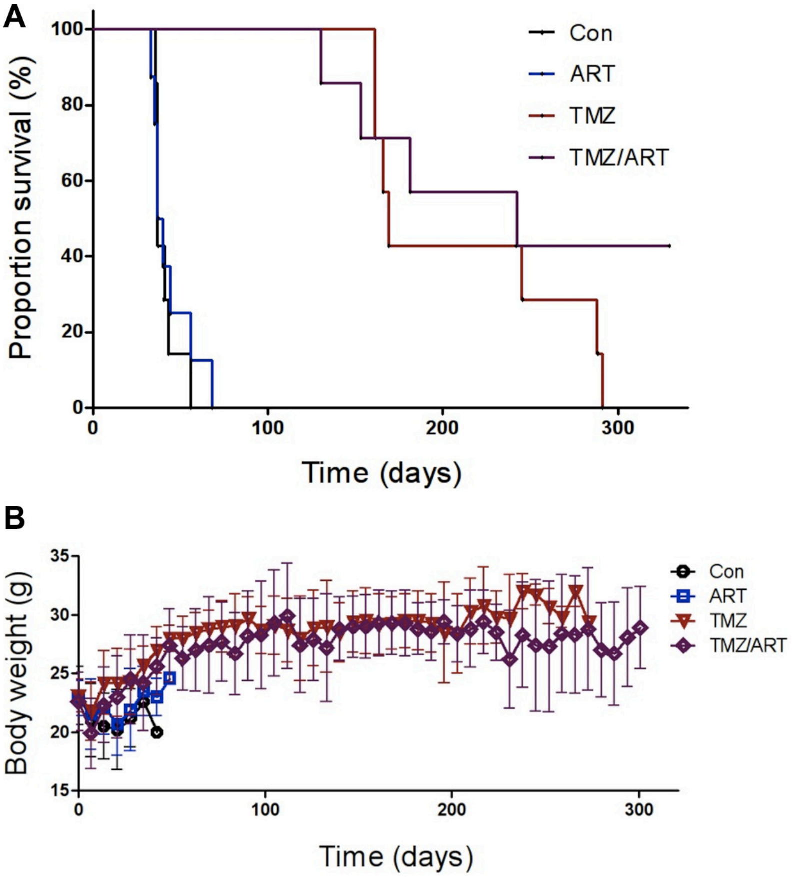

Supplementary Figure S1: Proportion of survival and body weight of mice bearing LN229 intracranial tumors. Tumor growth was monitored by MRT and verified by histology. Onset of treatment was three weeks after implantation of LN229 cells into the brain.Treatment occurred repeatedly with TMZ and ART as described in material and methods.
